# Correction: AI-Assisted chemical probe discovery for the understudied Calcium-Calmodulin Dependent Kinase, PNCK

**DOI:** 10.1371/journal.pcbi.1011672

**Published:** 2023-11-22

**Authors:** Derek J. Essegian, Valery Chavez, Rabia Khurshid, Jaime R. Merchan, Stephan C. Schürer

The first author, Derek J. Essegian, is incorrectly noted as the corresponding author. The correct corresponding author is Stephan C. Schürer (sschurer@miami.edu). The ORCID iD for Stephan C. Schürer is as follows: 0000-0001-7180-0978

There are errors in the author affiliations. The correct affiliations are as follows:

Derek J. Essegian **1**, Valery Chavez **2**, Rabia Khurshid **1**, Jaime R. Merchan **2,3**, Stephan C. Schürer *,1,3,4

**1** Department of Molecular and Cellular Pharmacology, University of Miami, Miami, FL, USA.

**2** Department of Medicine, University of Miami, Miami, FL, USA.

**3** Sylvester Comprehensive Cancer Center, University of Miami Miller School of Medicine, Miami, FL, USA.

**4** Institute for Data Science & Computing, University of Miami, Miami, FL, USA.
