# Supplementary material for: AI-Assisted chemical probe discovery for the understudied Calcium-Calmodulin Dependent Kinase, PNCK
Source: PLoS Comput Biol. 2023 May 26;19(5):e1010263. doi: 10.1371/journal.pcbi.1010263 (PMC10249896; doi:10.1371/journal.pcbi.1010263)
Supplement: S1 File — Fig A. Comparison of PNCK Schrödinger Prime homology model with AlphaFold model shows some differences but similar orientation with key interactions well aligned. A) PNCK Prime homology model (pink with bound ATP in yellow) aligned with AlphaFold model (blue with bound ATP in grey) in Schrödinger Maestro v3.0.137 using Protein Structure Alignment tool; shows considerable similarity in orientation of both the models; B) PNCK binding site shows key interactions are fairly aligned between both Prime and AlphaFold models; C) Hinge residues (Glu 91, Leu 92, Val 93) show similar orientation and interactions with ligand (ATP); D) DFG residues (Asp 157, Phe 158, Gly 159) show slight difference in orientation; E) Gatekeeper (Met 90) shows similar orientation; F) Key residue, Lys 44, shows similar orientation and interaction with the ligand (ATP); G) Key residues Asn 141 shows slightly different orientation and makes interaction with ligand in AlphaFold model; H) α–c loop is somewhat different among the two models; I) 2D interactions of PNCK AlphaFold model with bound ATP; J) 2D interactions of PNCK Schrödinger Prime model with bound ATP. Fig B. PNCK Shrödinger Prime homology model conformation identification using KinCoRe. Fig C. Molecular Dynamics (MD) Analysis of 3 PNCK Homology Models. (A-C), Molecular Dynamic RMSD analysis of protein (C-alpha) and ligand fit on protein (4FG7, 4FG8, 4FG9). (D-F) Interaction Fraction of ATP to Residues in PNCK Active Site (4FG7, 4FG8, 4FG9). Fig D. SwissModel Ramachandran Plots for PNCK Homology Models. A) 4FG7 Homology Model, B) 4FG7 Original, C) 4FG8 Homology Model, D) 4FG8 Original, E) 4FG9 Homology Model, F) 4FG9 Original. Fig E. ADP-Kinase GLO Results from Naïve Bayesian Classifier Campaign. Fig F. UM_228 Binding Mode Hypothesis After MD, 4FG8 Model. A) 2D representation, B) 3D Representation C) 3D Surface Representation of Binding Pocket. Fig G. Molecular Dynamics Analysis of UM_228. A) Protein-Ligand RMSD plot of 500ns MD simulation [file pcbi.1010263.s004.pdf]

Fig A in S1 File

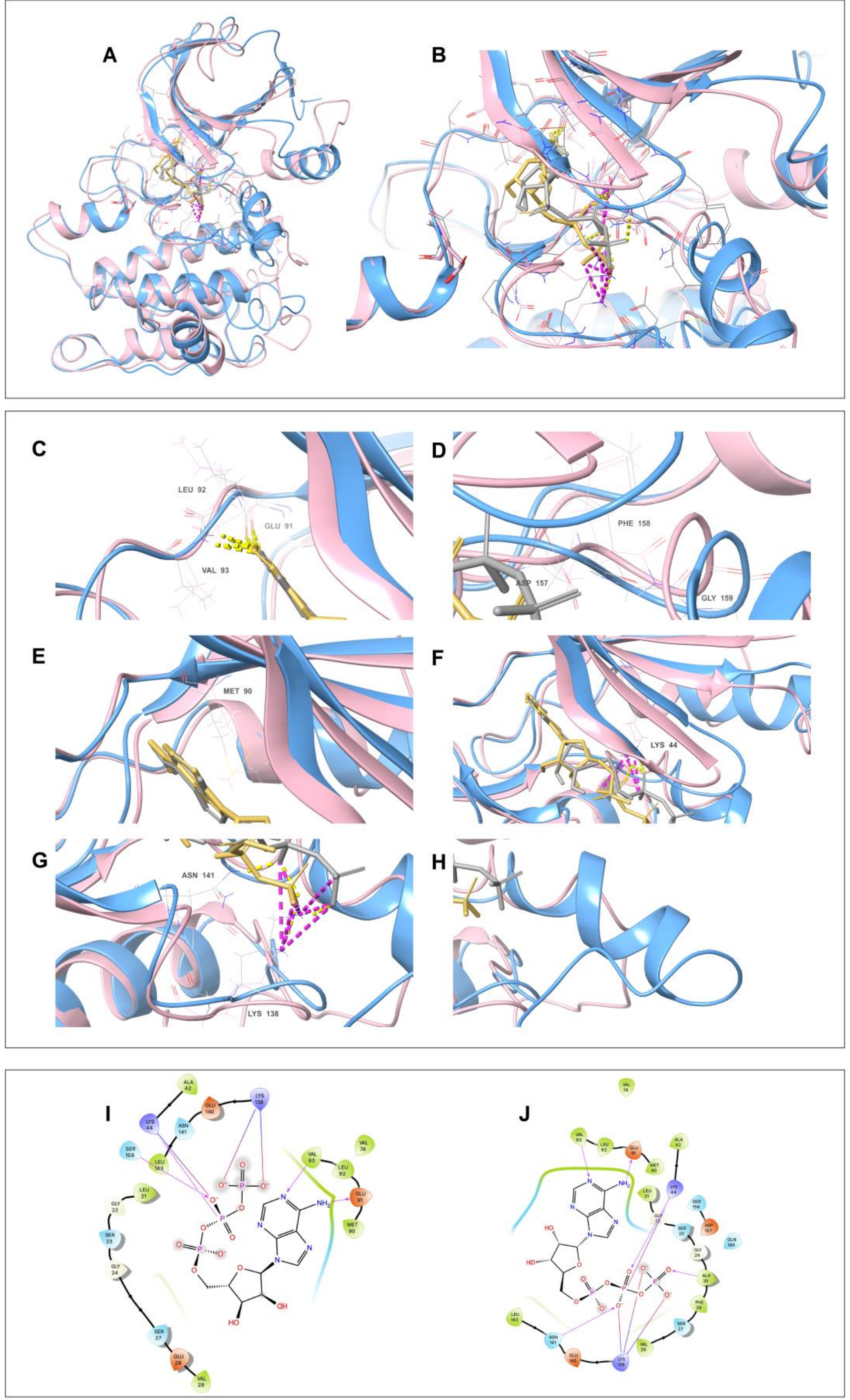

Fig B in S1 File

PNCK Prime Model	Chain	Group	Spatial label	Dihedral label	Ligand	X-DFG Φ, Ψ	DFG-Asp Φ, Ψ	DFG-Phe Φ, Ψ, χ1
4FG7_homology	A	CAMK	DFGin	BLAminus	None	[S156] -142.95, 170.14	[D157] 75.46, 82.11	[F158] -87.33, -2.77, 286.48
4FG8_homology	A	CAMK	DFGinter	Unassigned	None	[S156] -138.31, 63.37	[D157] -171.92, 162.7	[F158] -138.94, 12.83, 305.44
4FG9_homology	A	CAMK	DFGinter	Unassigned	None	[S156] -61.88, -76.44	[D157] -71.3, 111.66	[F158] -83.98, 46.95, 48.4

Fig C in S1 File

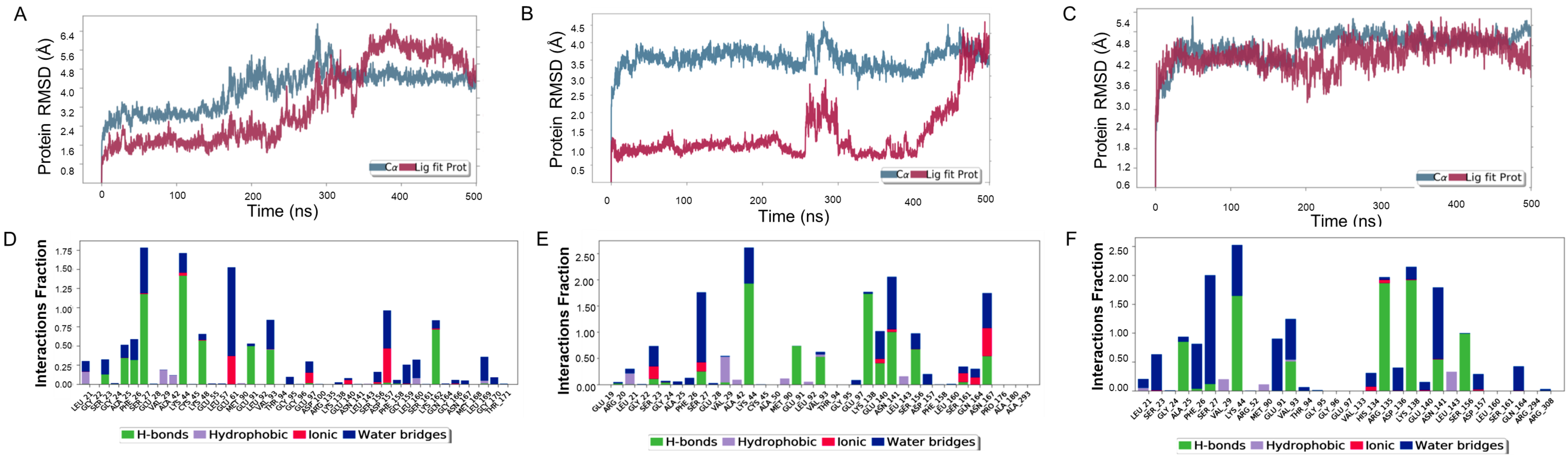

Fig D in S1 File

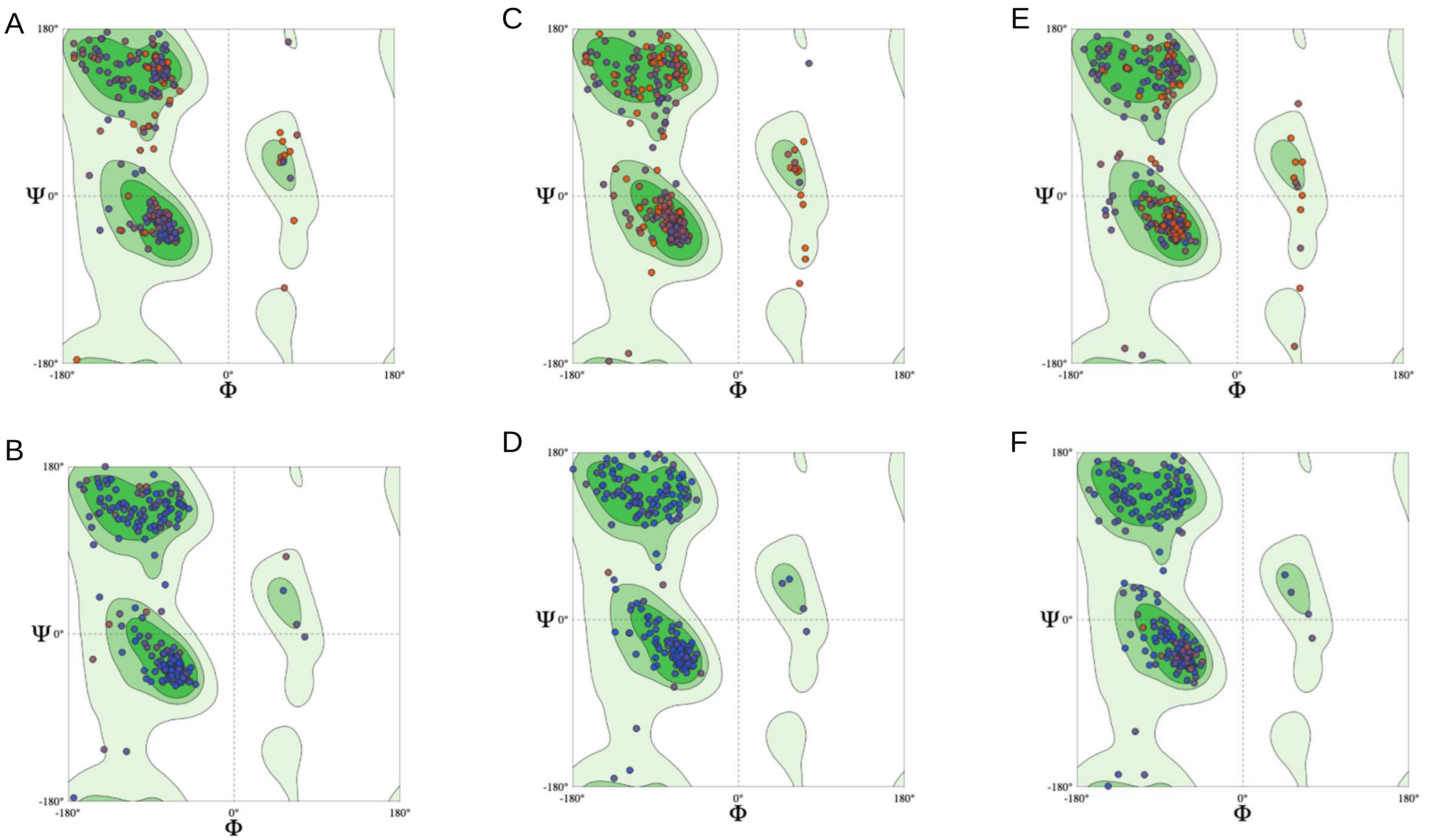

Fig E in S1 File

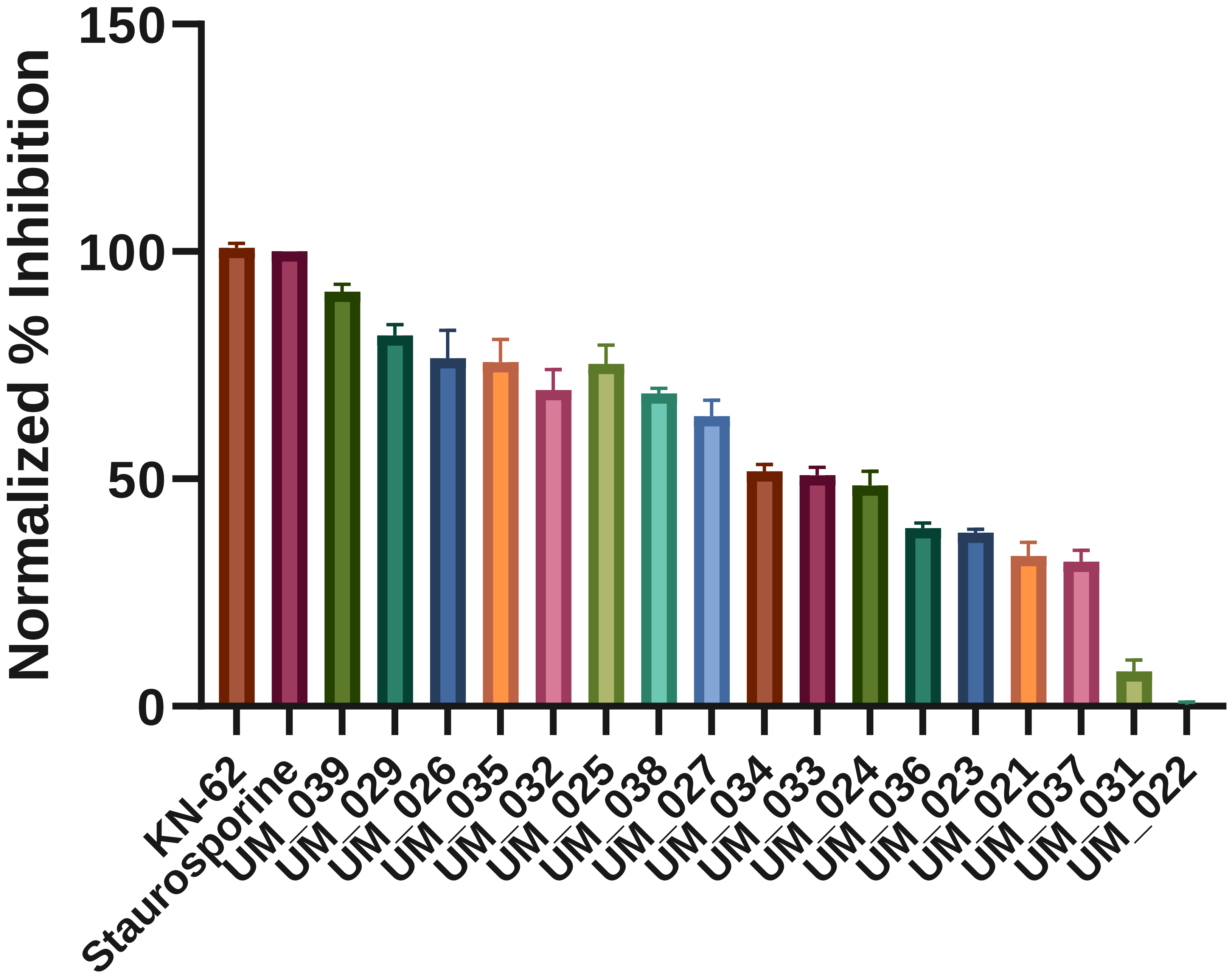

Fig F in S1 File

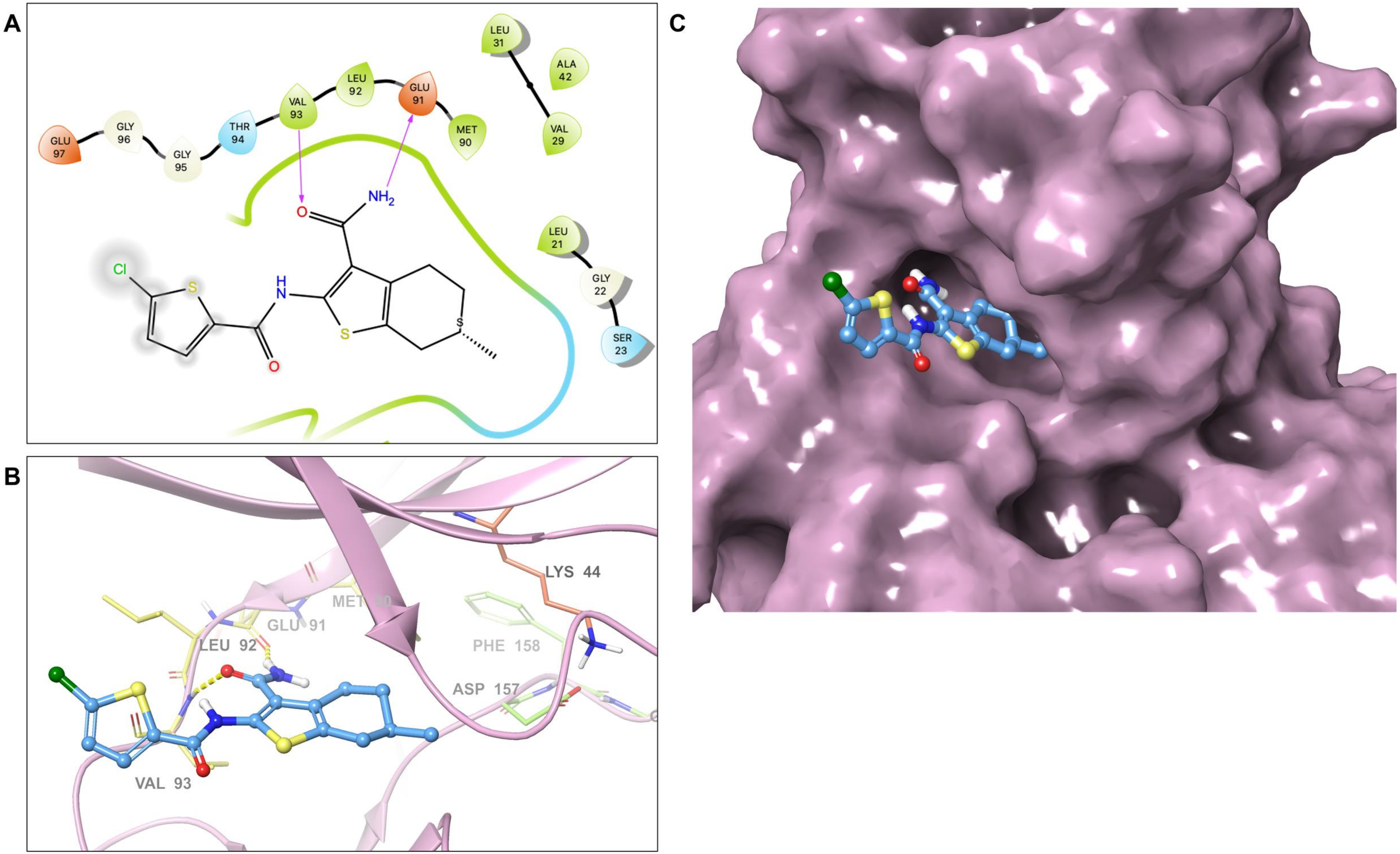

Fig G in S1 File

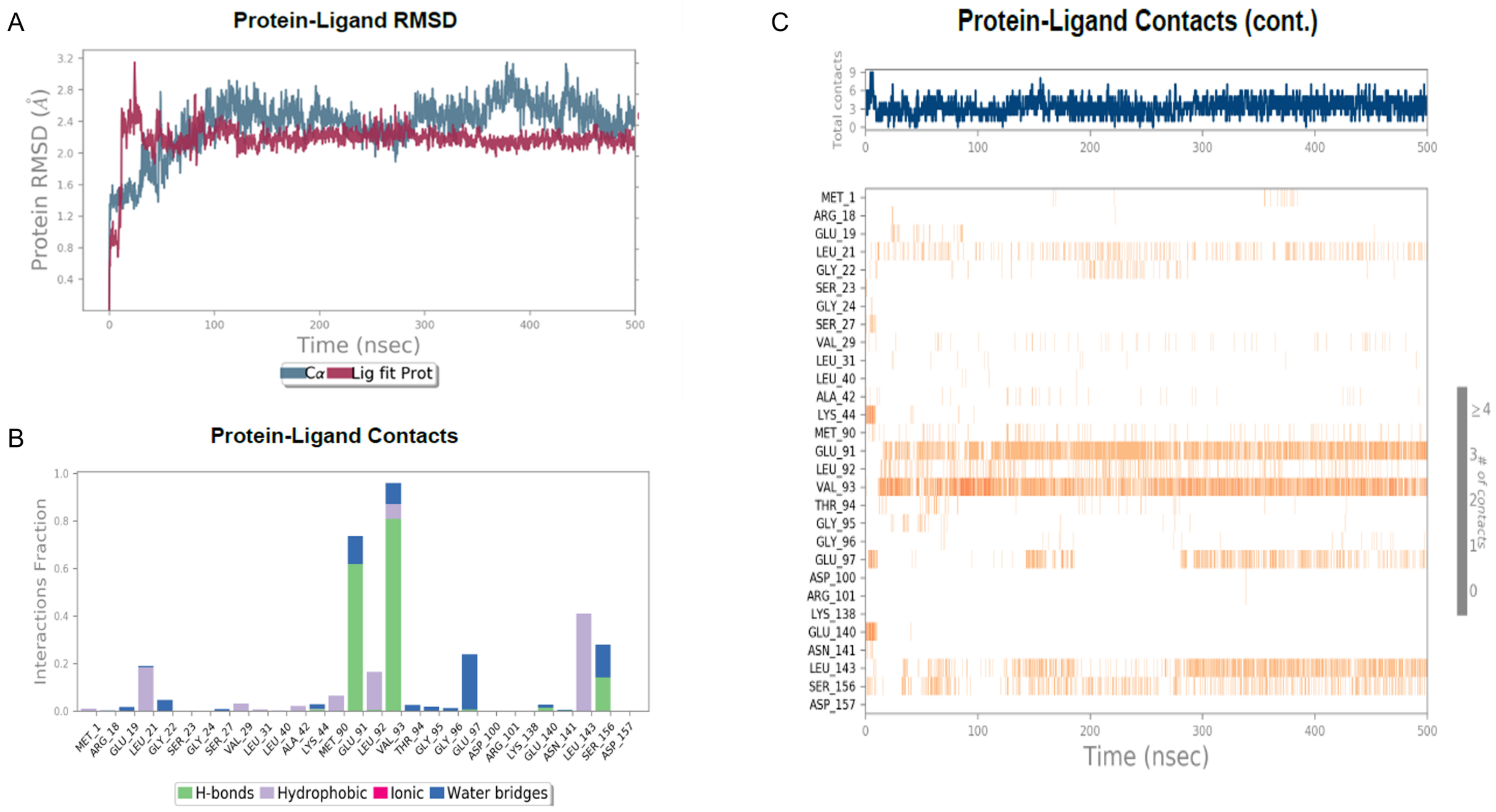

Fig H in S1 File

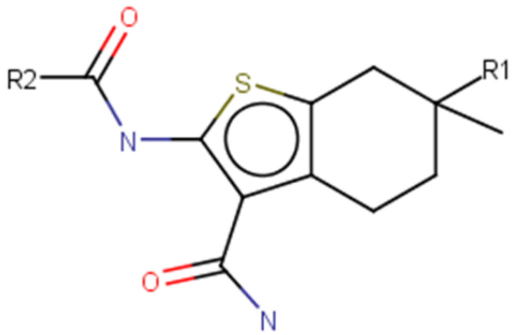

UM ID	R1	R2	IC50 µM
**UM_228**	—H	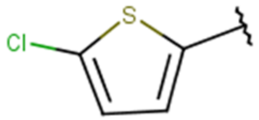	8.32 (+/- 1.32)
**UM_250**	—H	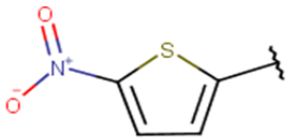	>> 10
**UM_251**	—H	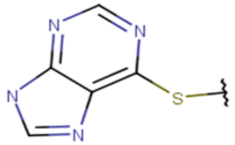	2.54 (+/- 1.97)
**UM_252**	—H	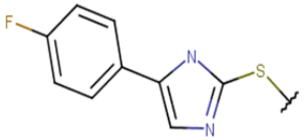	1.09 (+/- 0.88)
**UM_255**	—H	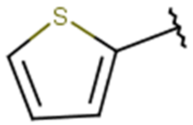	>> 10
**UM_260**	—CH_3	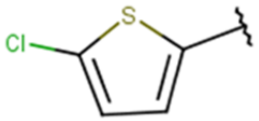	>> 10

Fig I in S1 File

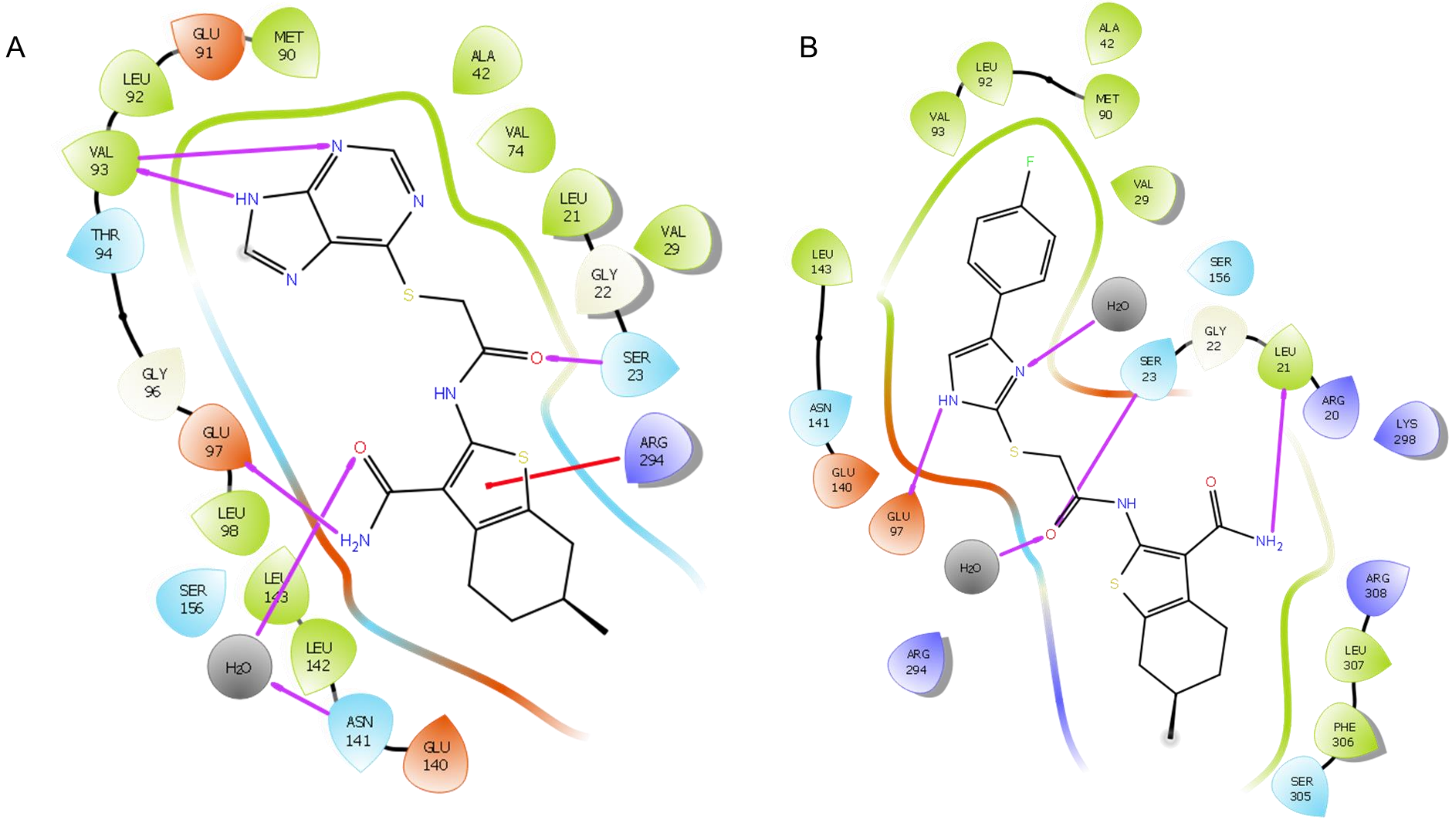

Fig J in S1 File

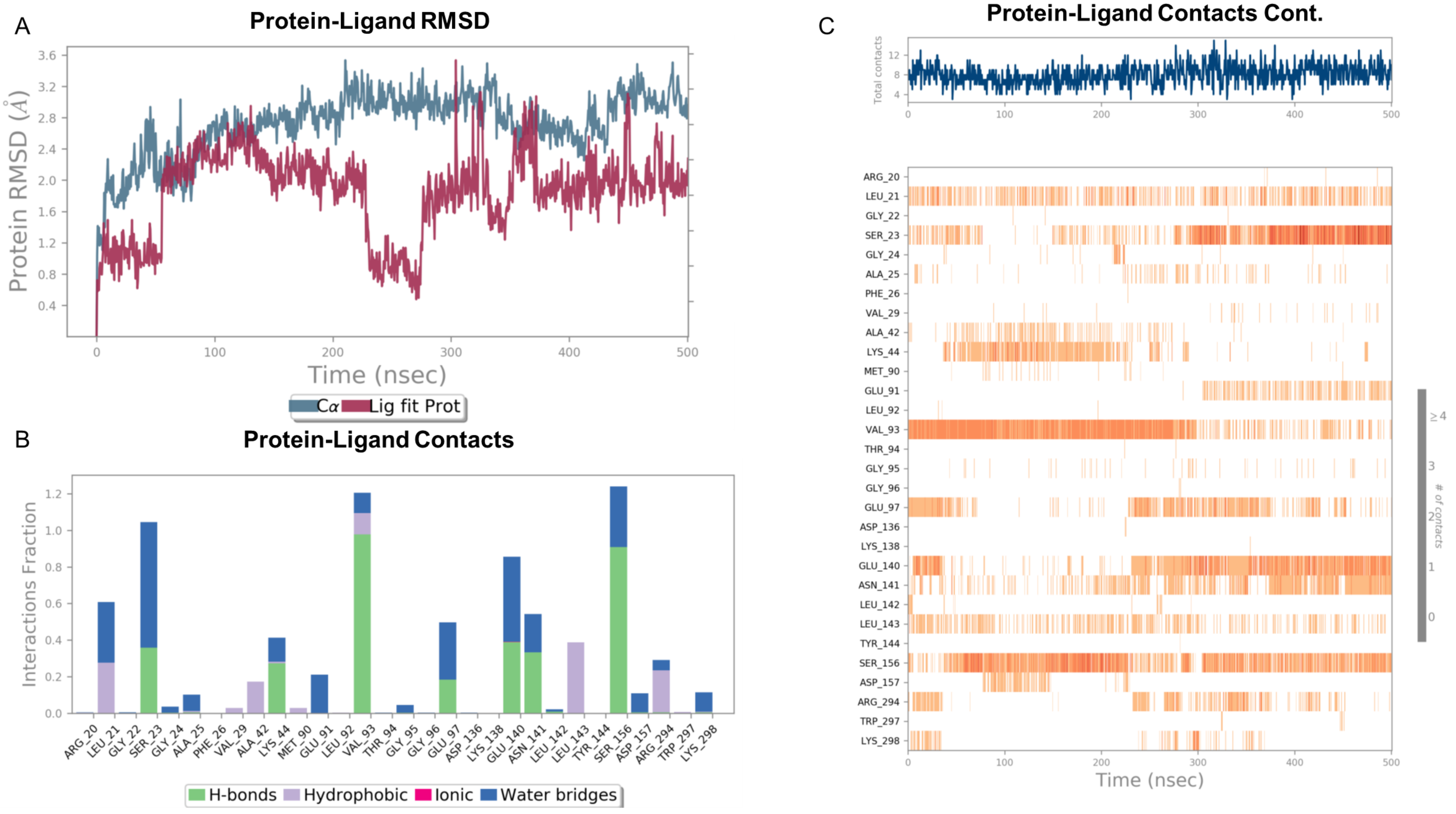

Fig K in S1 File

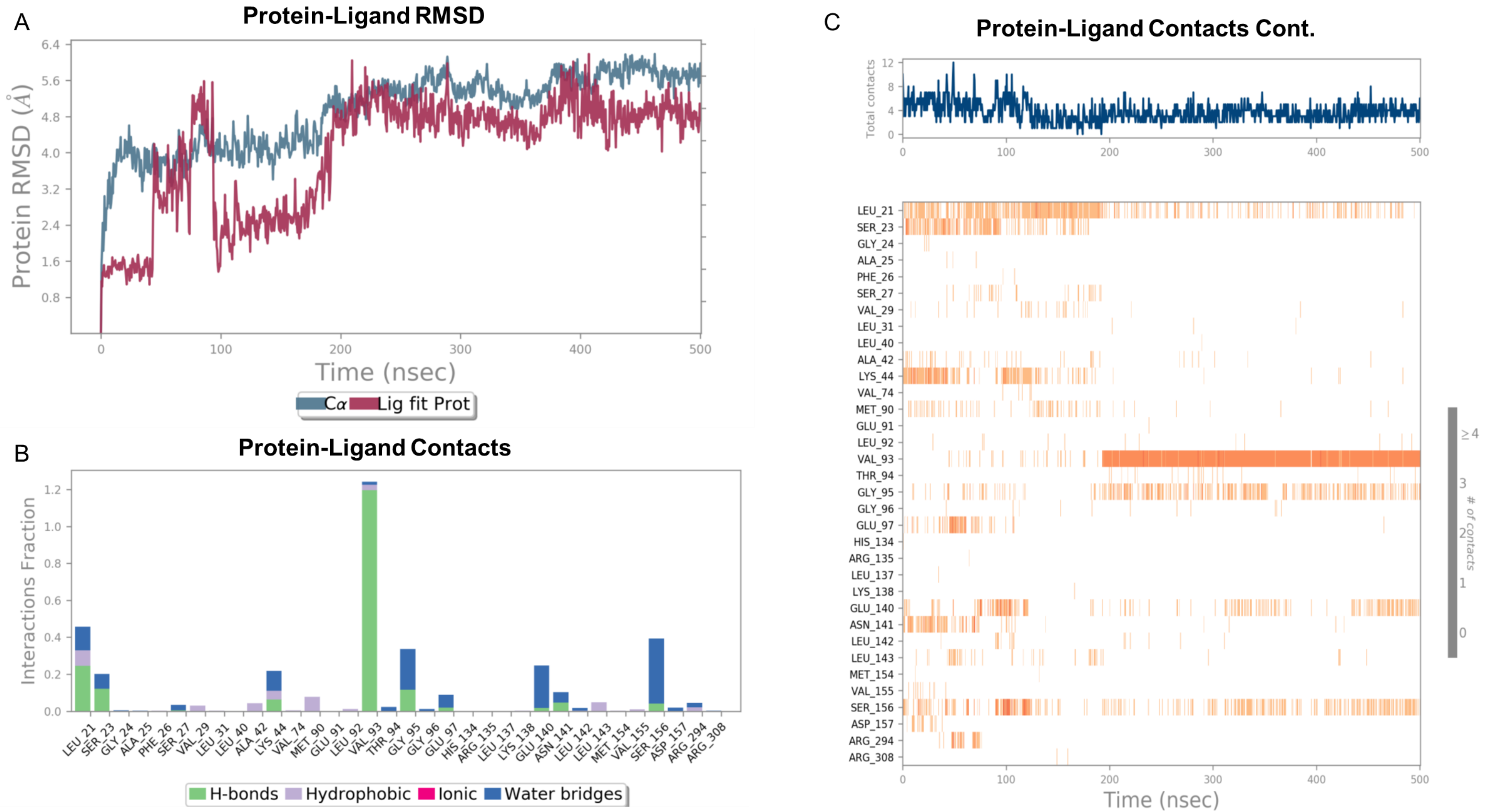

Fig L in S1 File

A

B

C

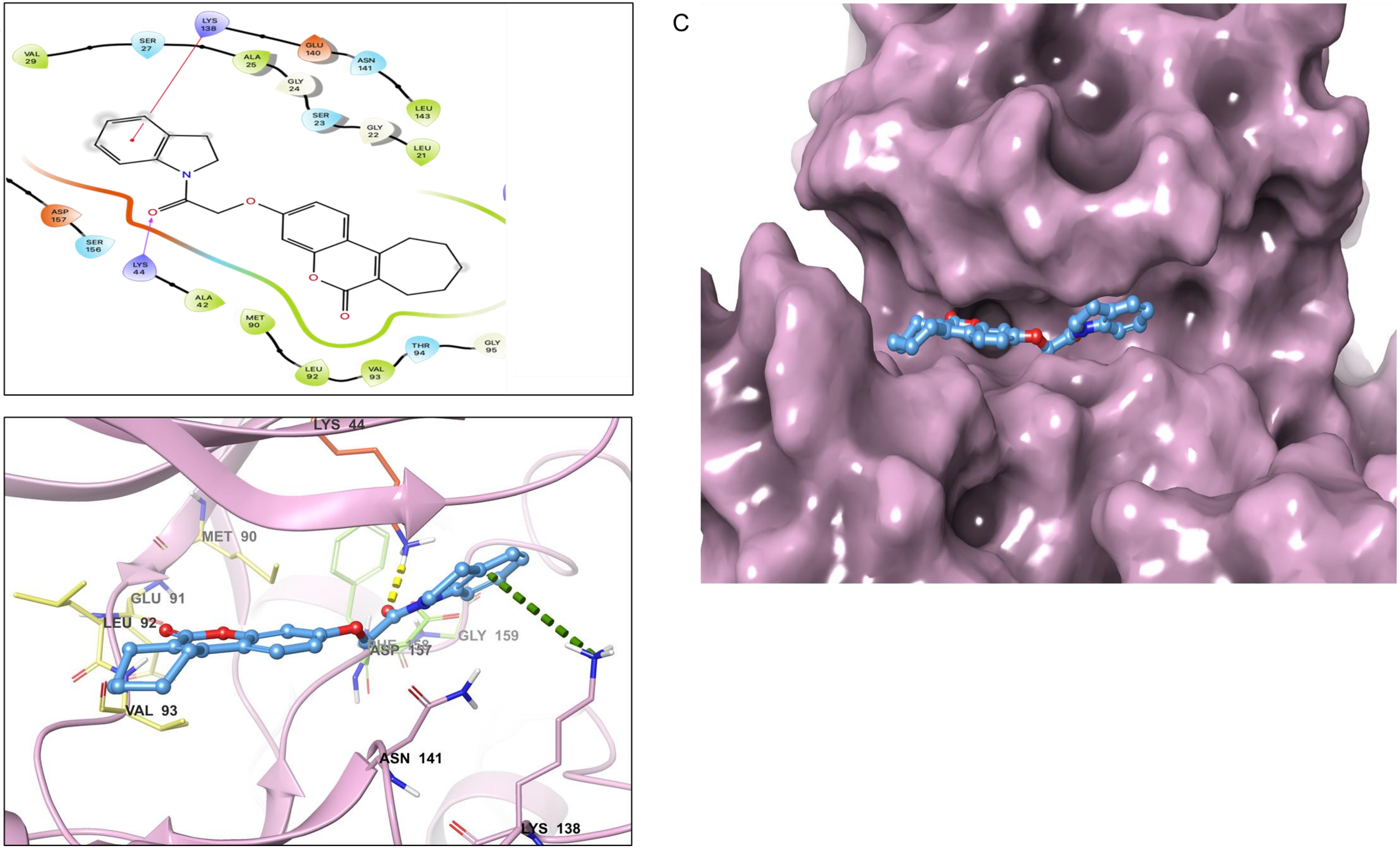

Fig M in S1 File

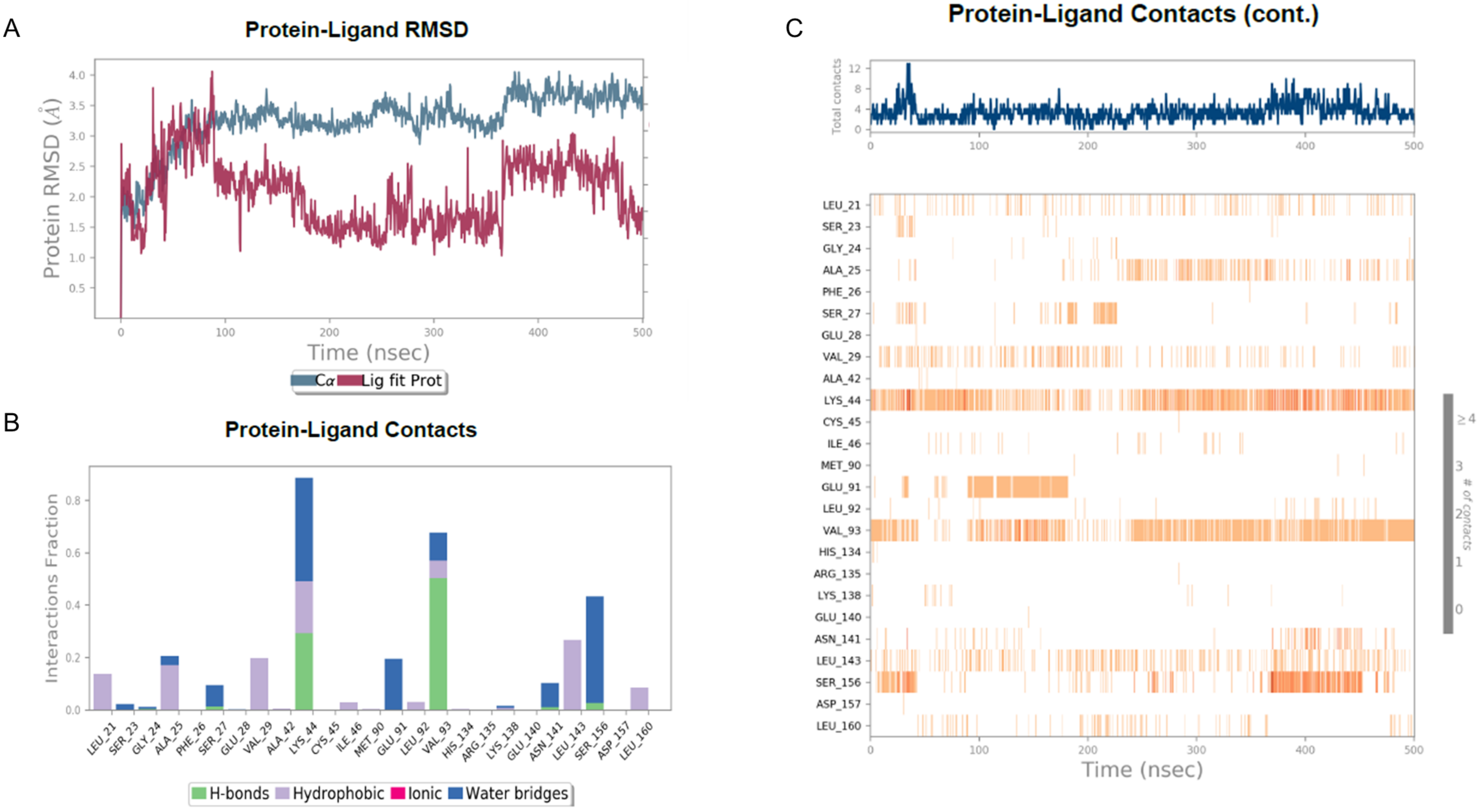

Fig N in S1 File

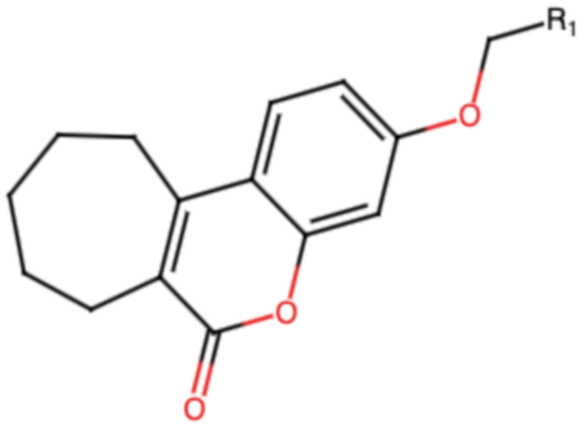

UM ID	R1	IC50 µM
UM_213	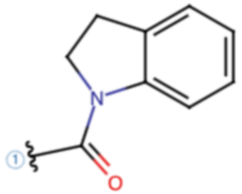	9.49 (+/- 3.58)
UM_258	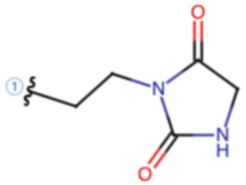	>>10
UM_259	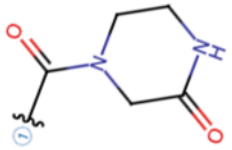	2.90 (+/- 0.99)

Fig O in S1 File

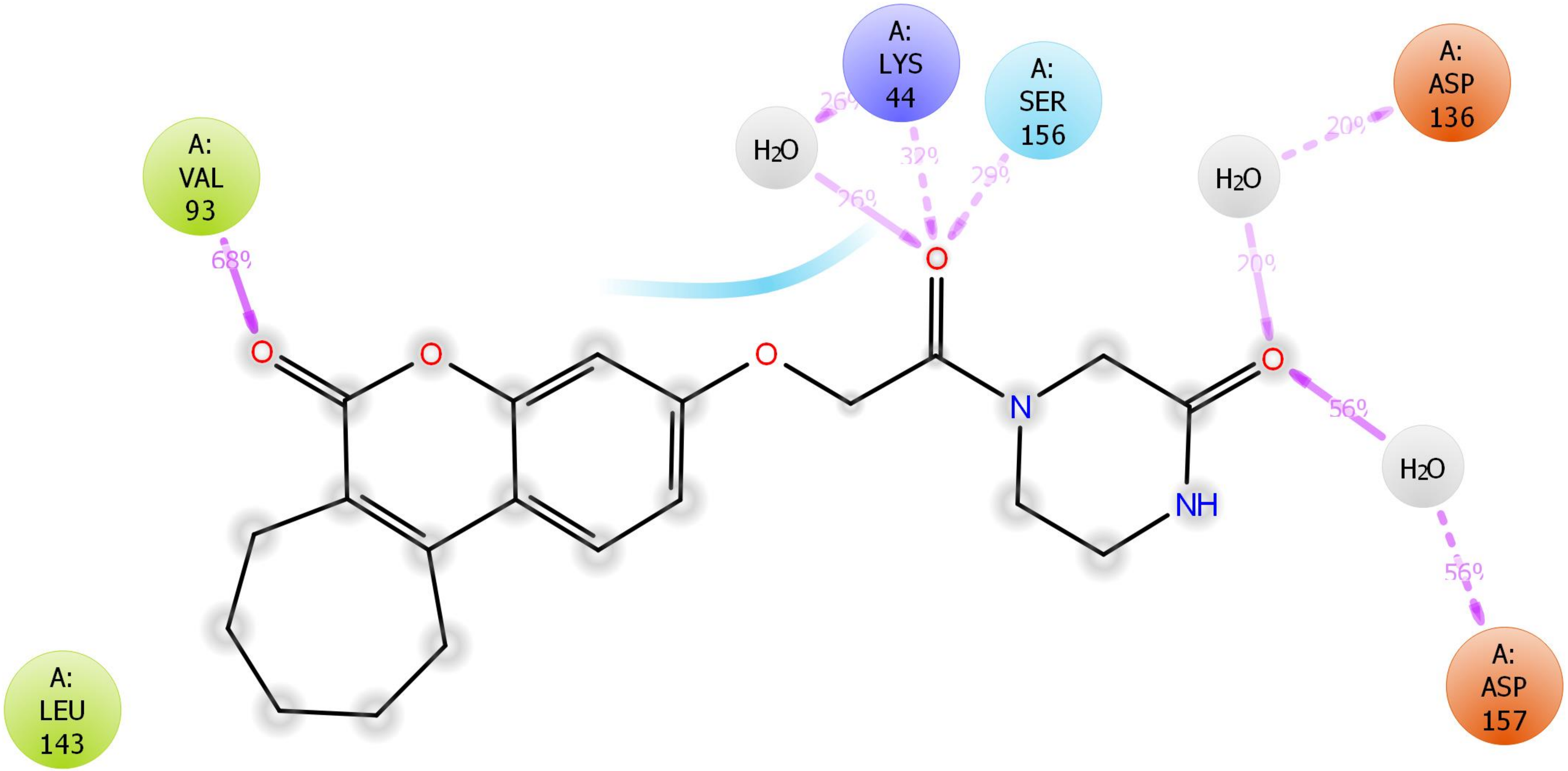

Fig P in S1 File

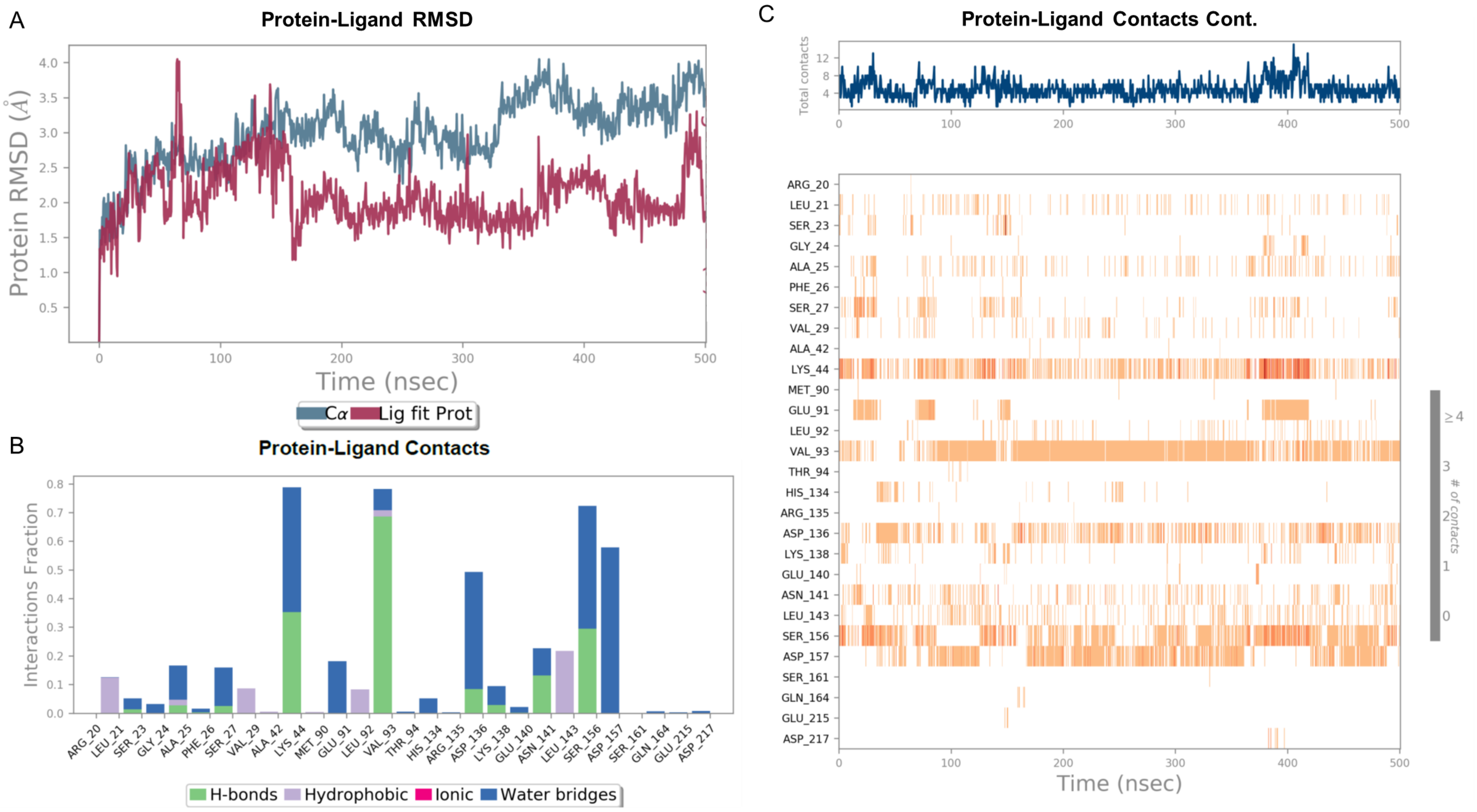

Fig Q in S1 File

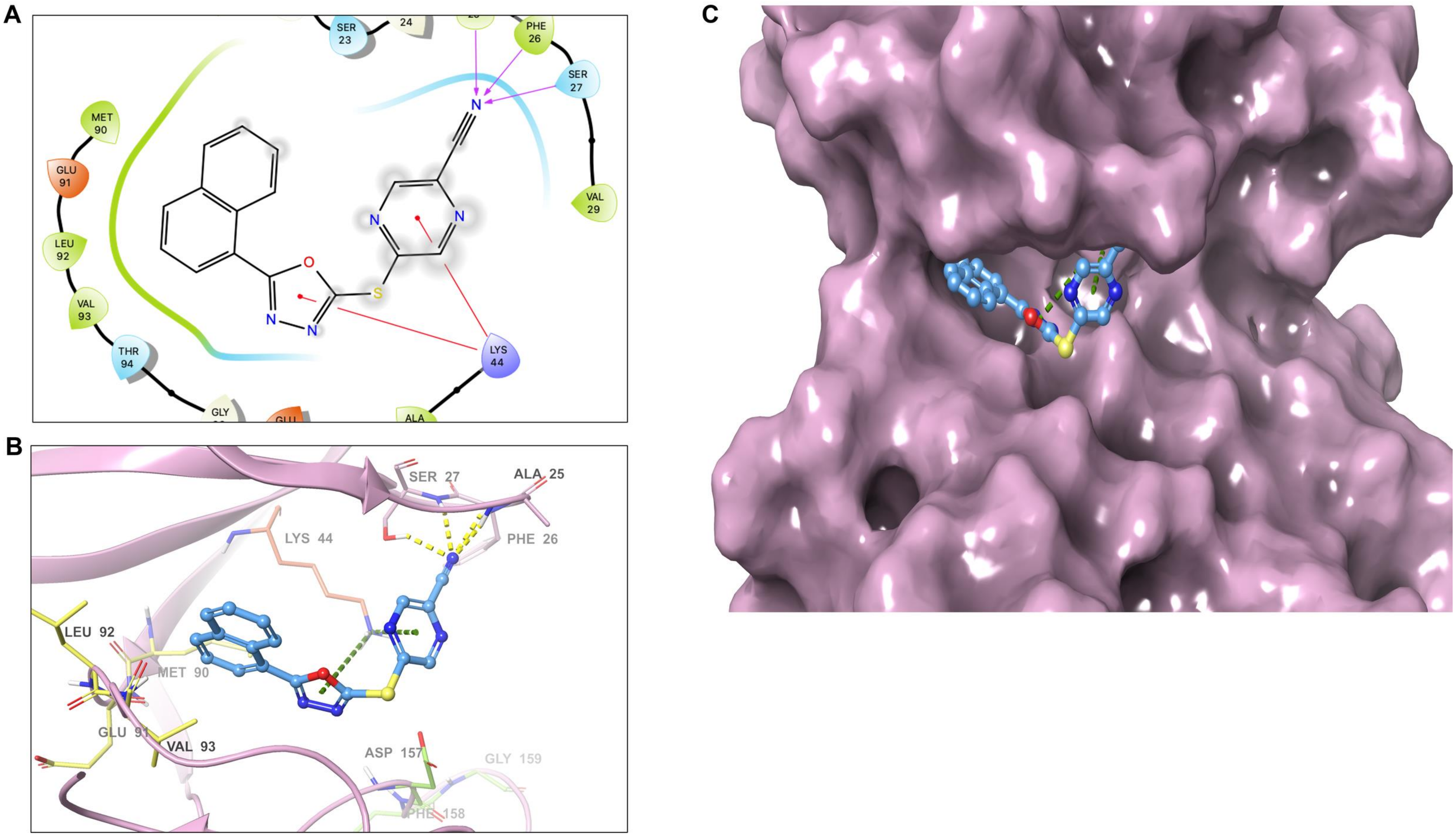

Fig R in S1 File

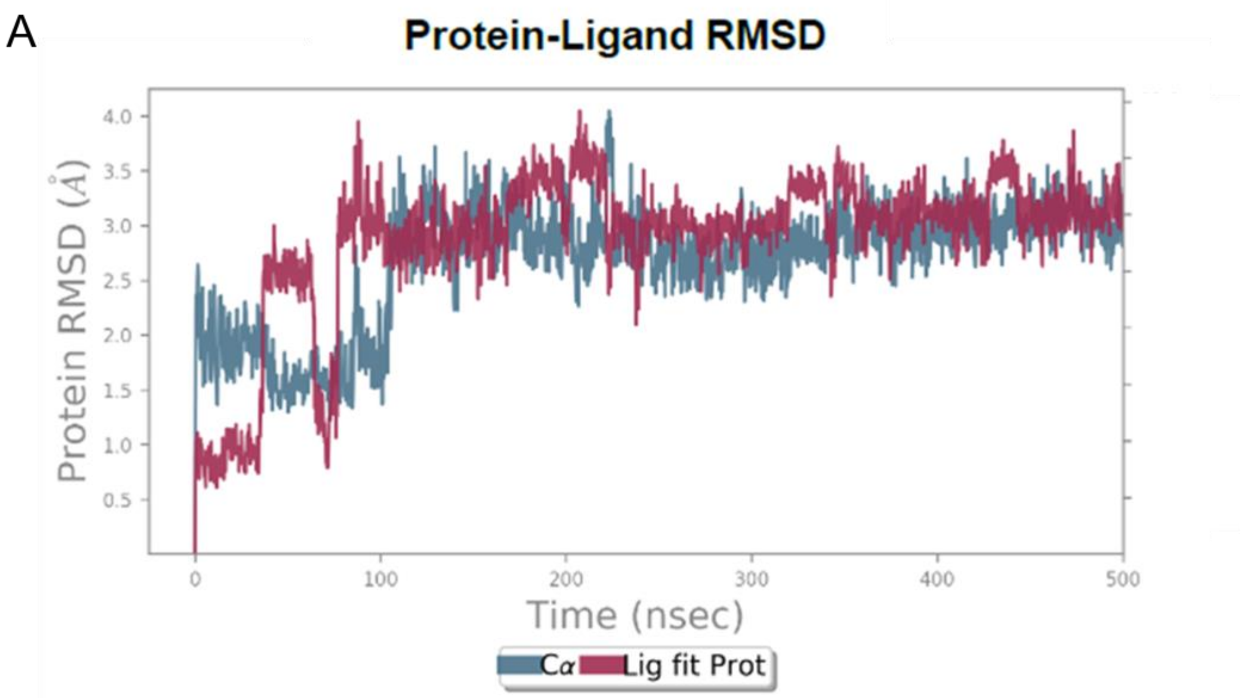

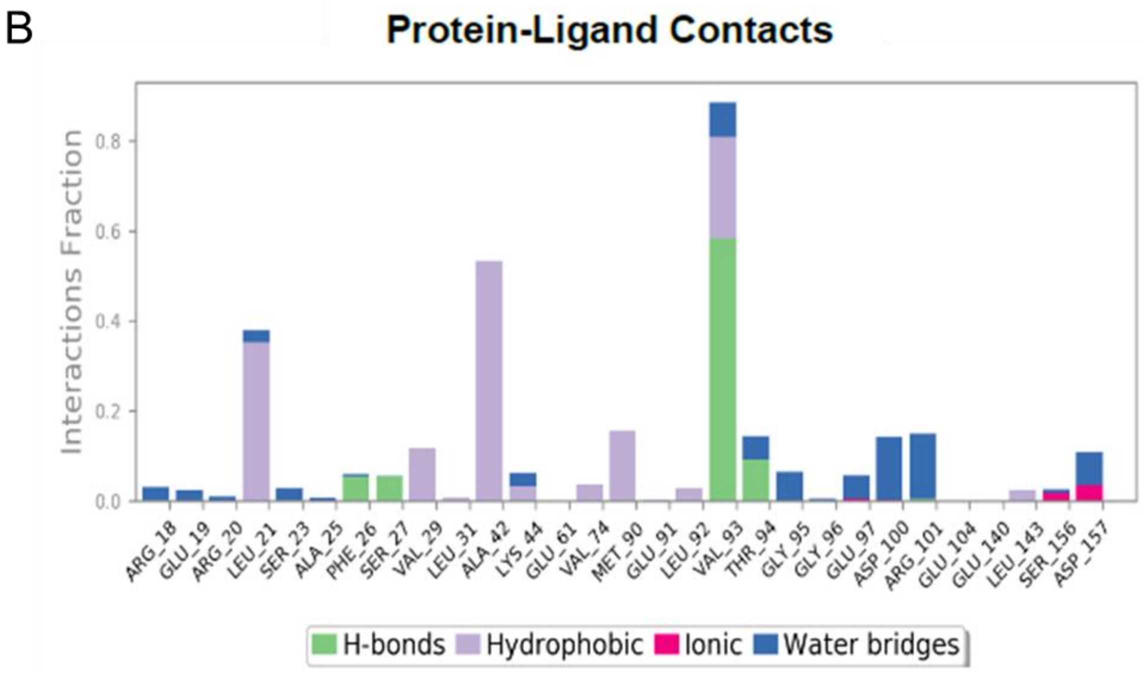

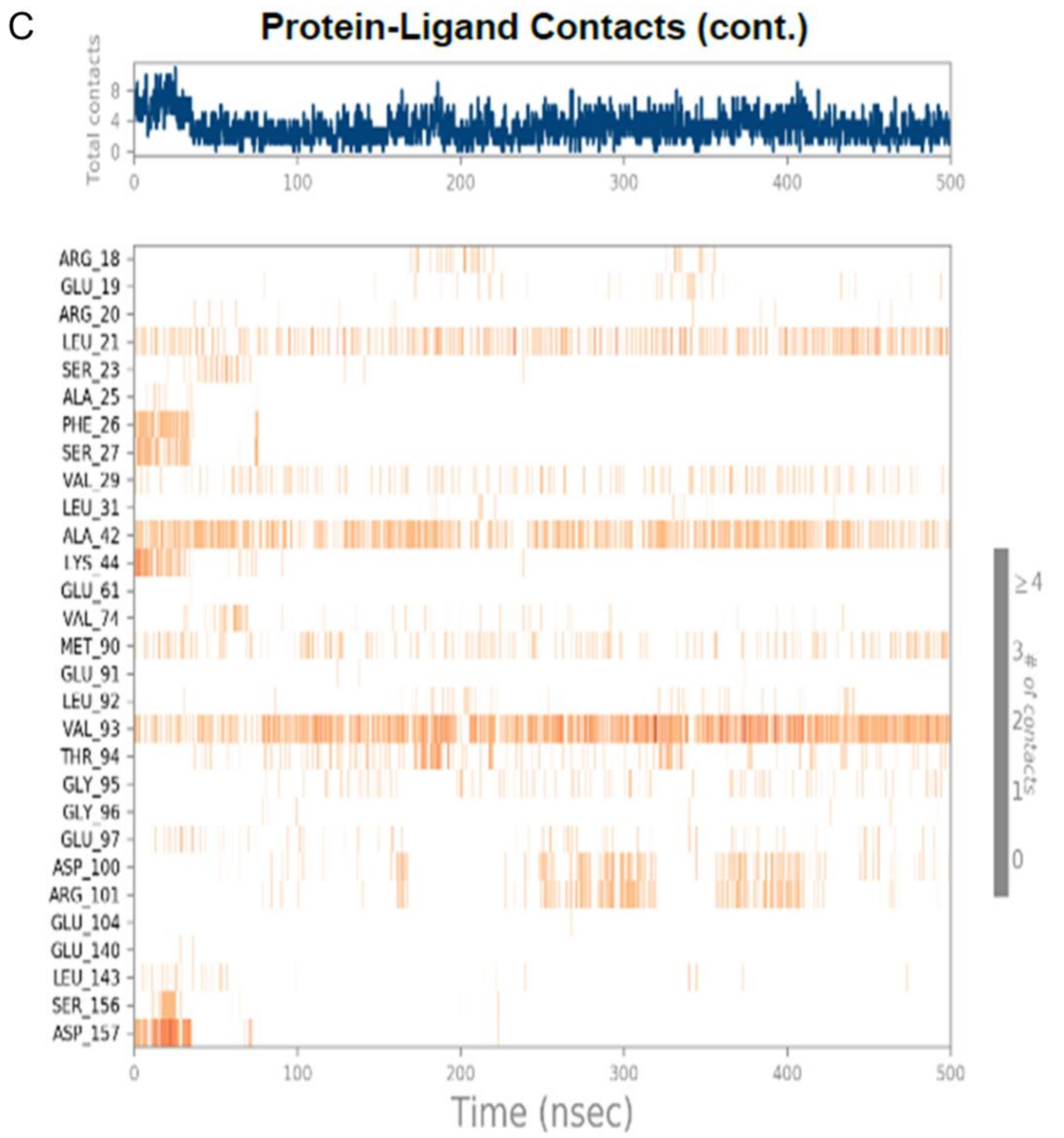

Fig S in S1 File

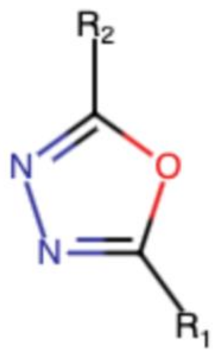

UM ID	R1	R2	IC50 µM
**UM_195**	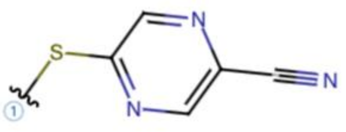	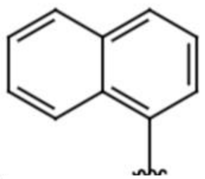	3.36 (+/- (0.91)
**UM_253**	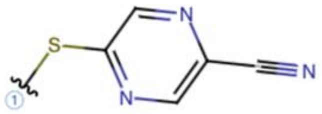	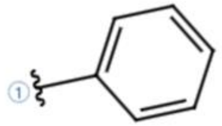	>>10
**UM_254**	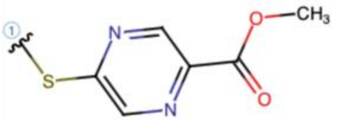	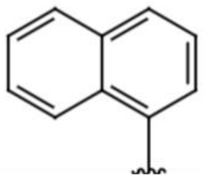	2.68 (+/- 1.16)
**UM_256**	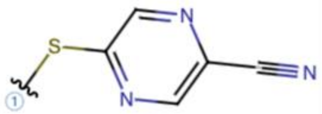	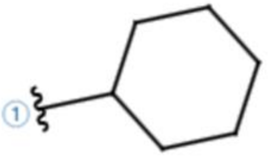	>>10
**UM_265**	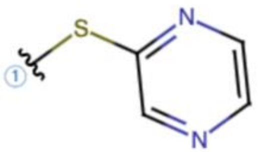	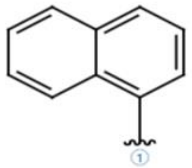	>>10

Fig T in S1 File

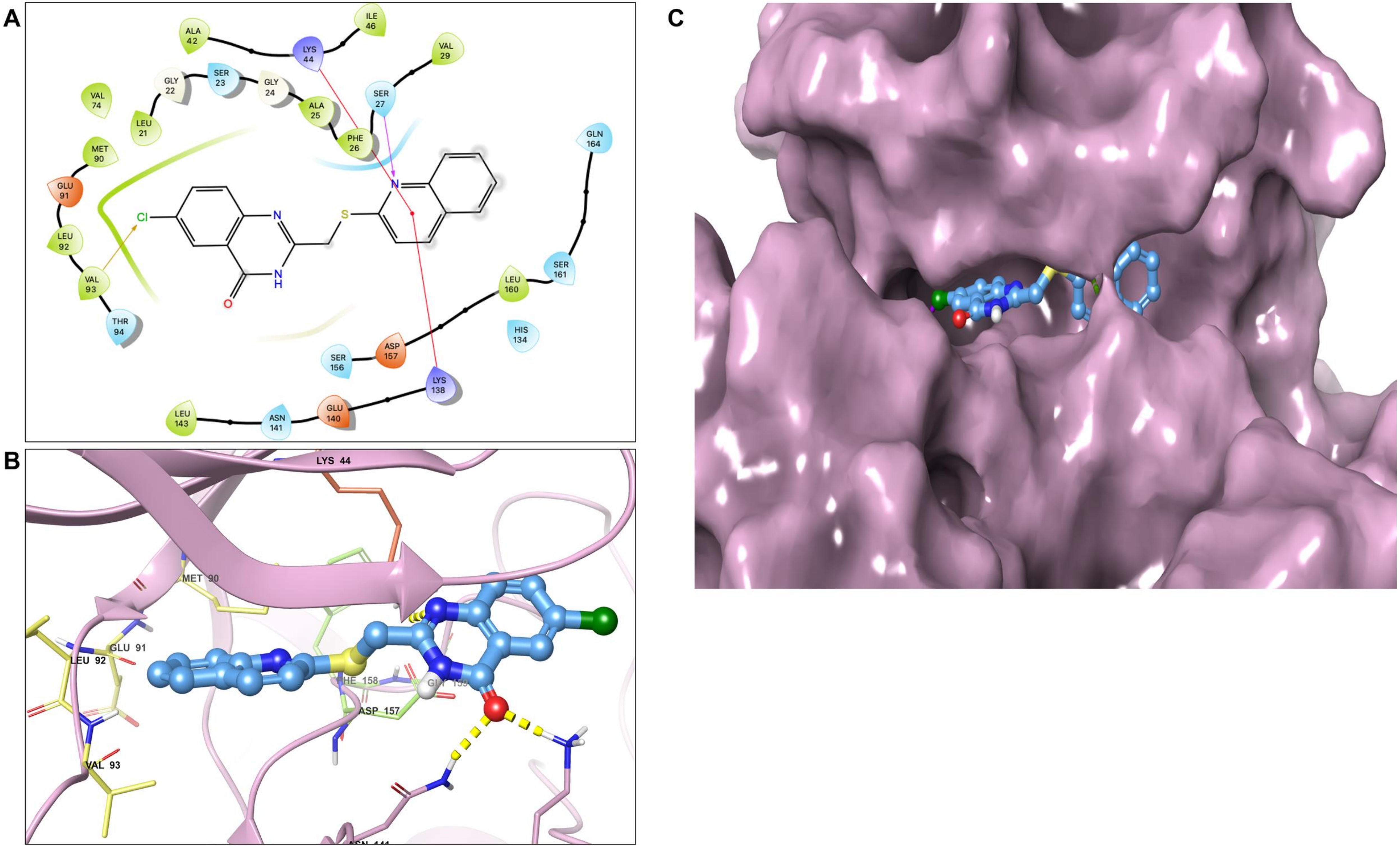

Fig U in S1 File

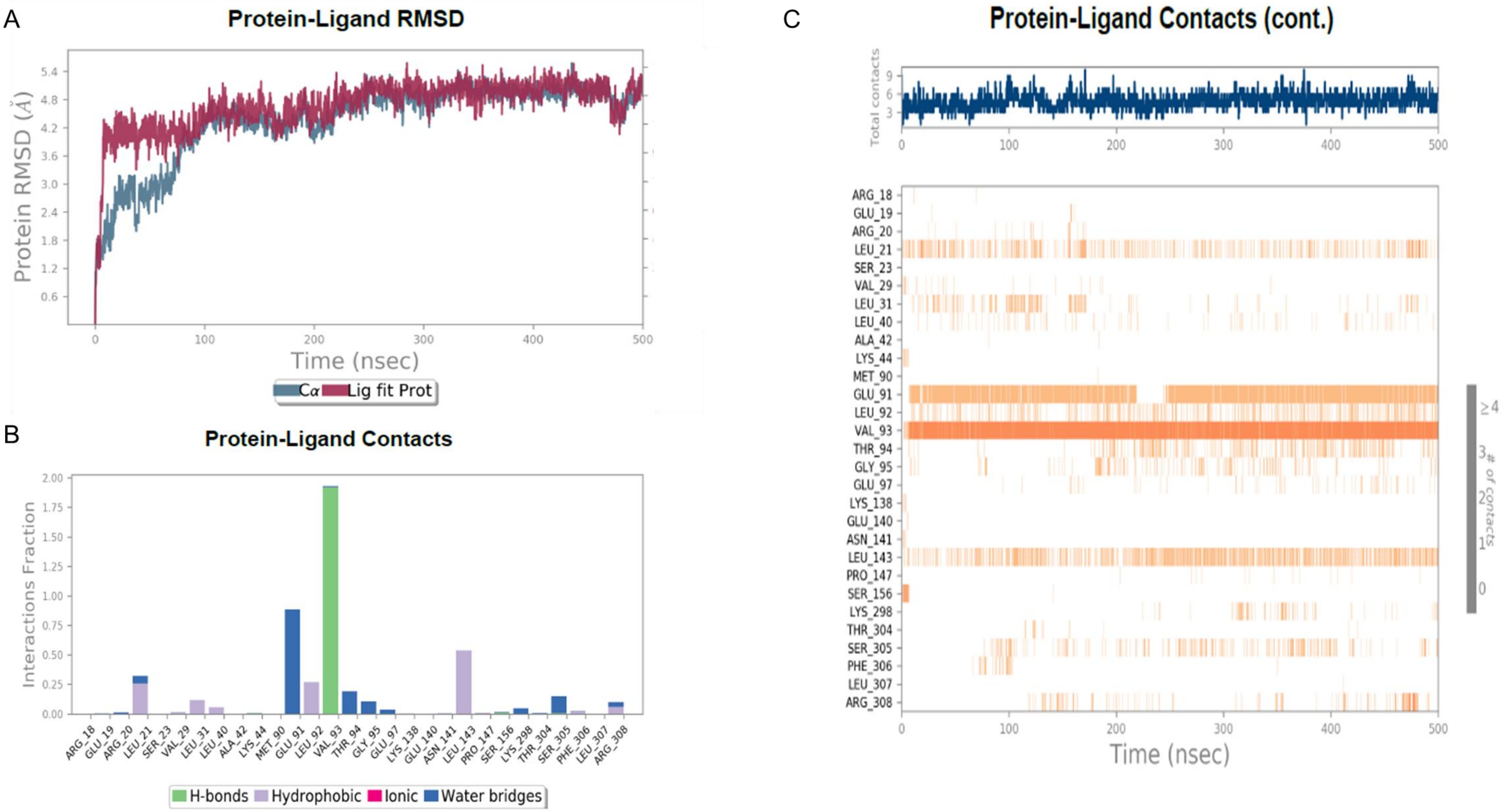

Fig V in S1 File

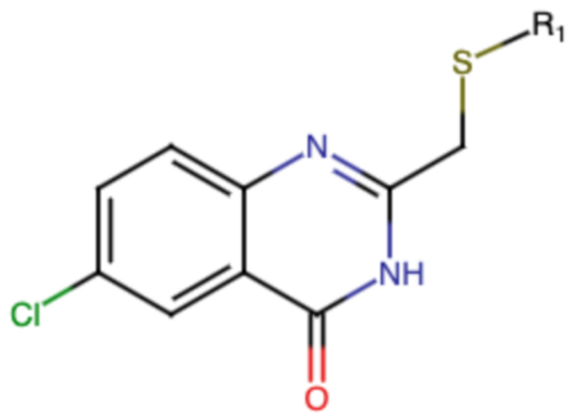

UM ID	R1	IC50 μM
**UM_210**	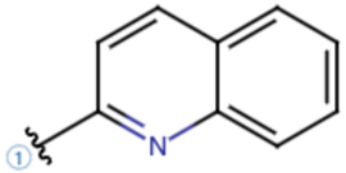	3.36 (+/- 2.19)
**UM_278**	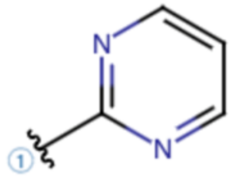	>>10
**UM_279**	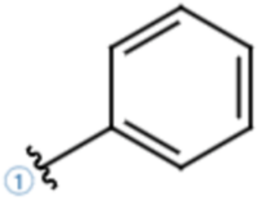	2.68 (+/- 0.86)
**UM_280**	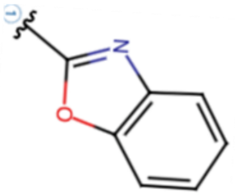	>>10

Fig W in S1 File

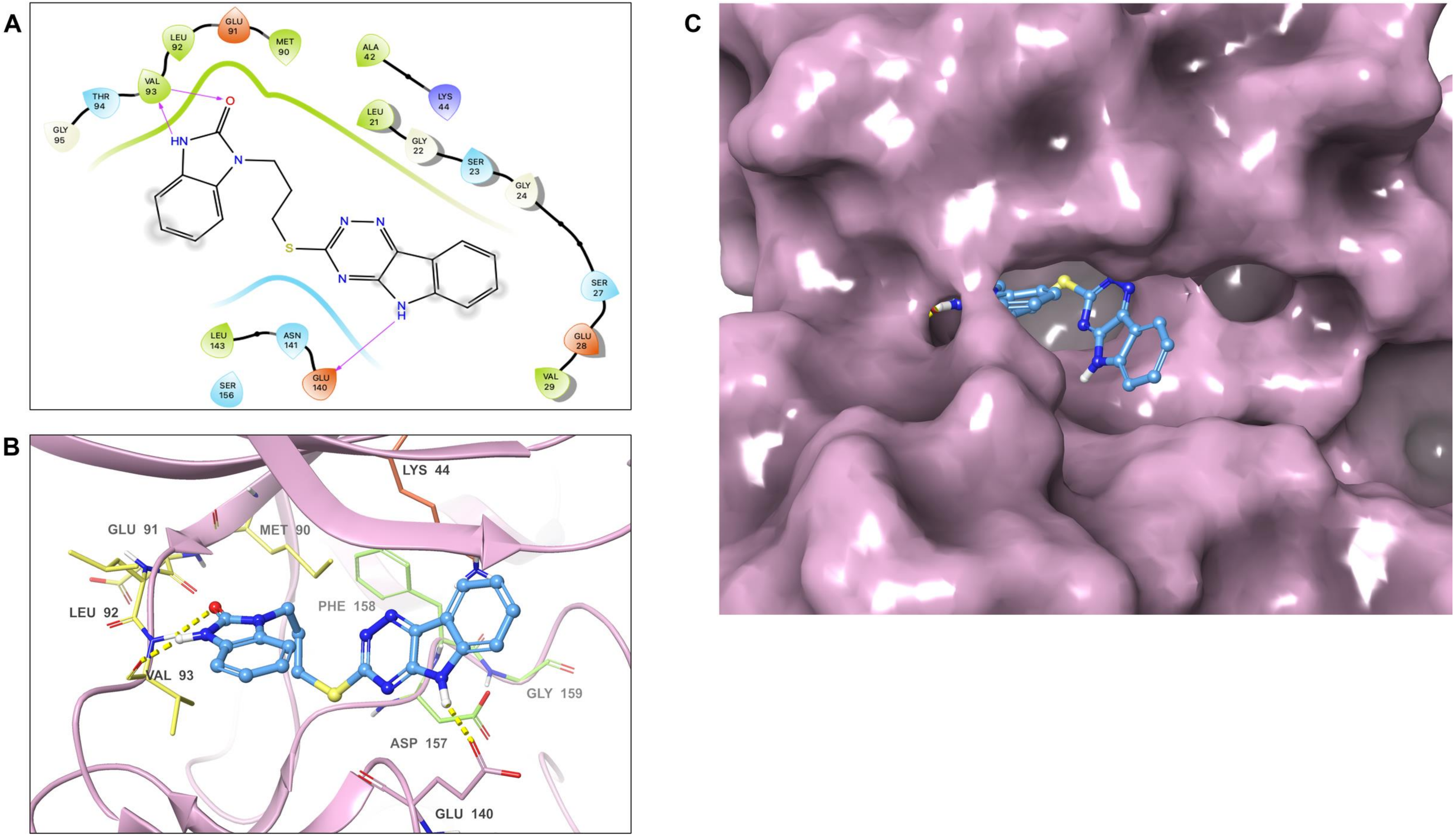

Fig X in S1 File

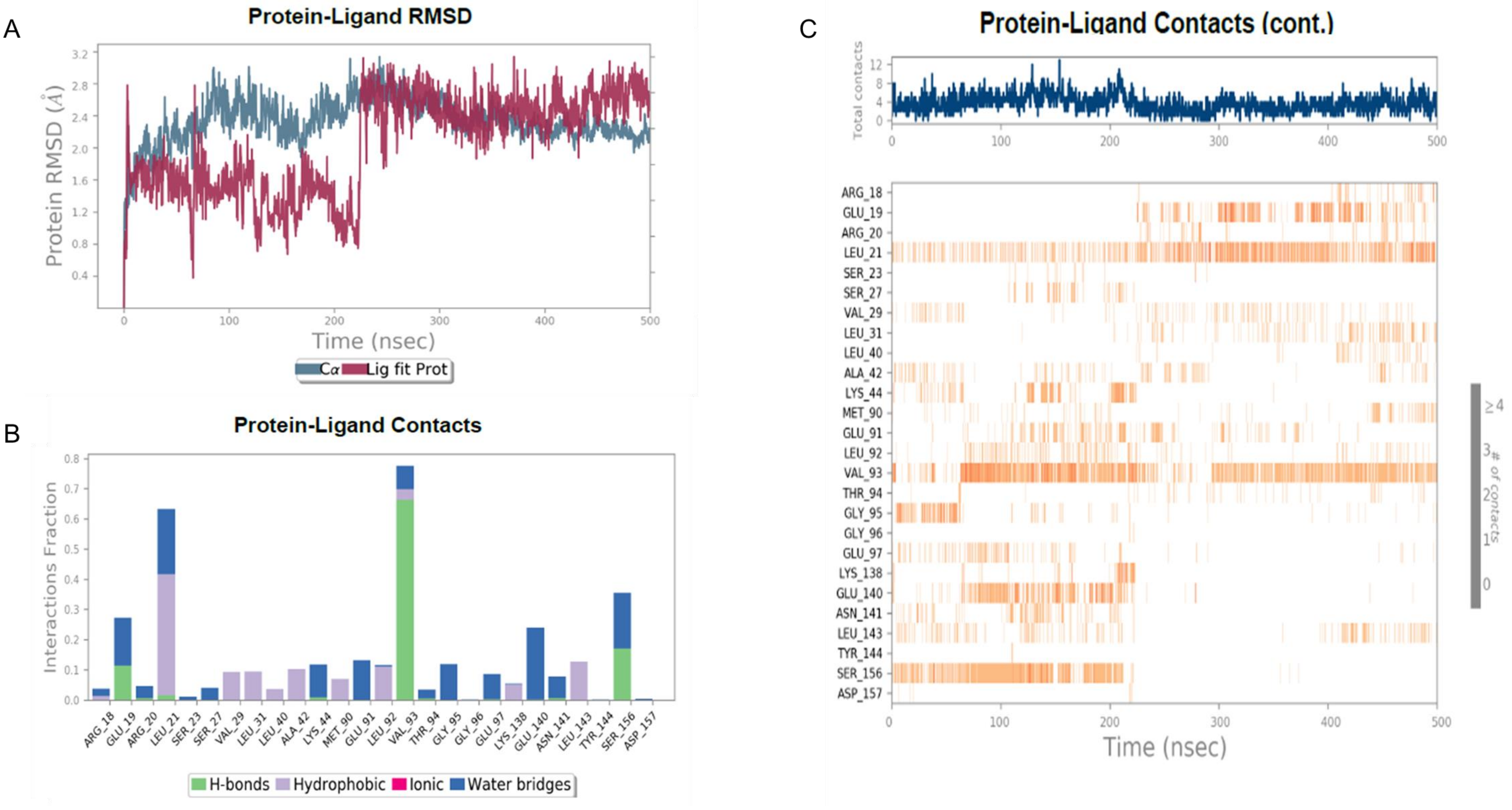

Fig Y in S1 File

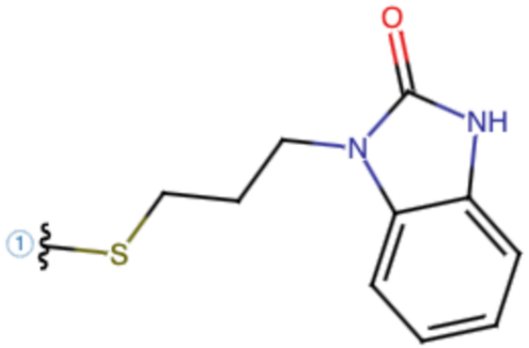

UM ID	R1	IC50 µM
**UM_037**	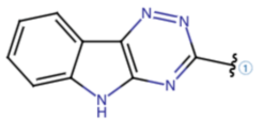	8.78 (+/- 1.32)
**UM_273**	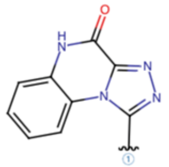	>>10
**UM_274**	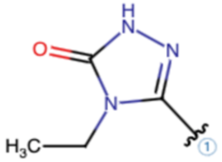	10.30 (+/- 3.76)
**UM_275**	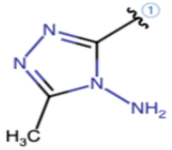	>>10
**UM_276**	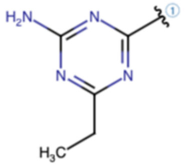	>>10
**UM_277**	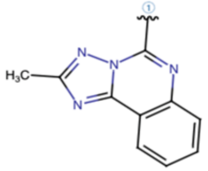	16.1 (+/- 6.32)
